# Supplementary material for: Estimated Phytate Intake Is Associated with Bone Mineral Density in Mediterranean Postmenopausal Women
Source: Nutrients. 2023 Apr 6;15(7):1791. doi: 10.3390/nu15071791 (PMC10097286; doi:10.3390/nu15071791)
Supplement: Supplementary file 1 [file nutrients-15-01791-s001.zip › Supplementary Table S1.pdf]

Supplementary Table S1. Odds Ratio and 95% CIs between Low Bone Mineral Density (T-score ≤ -1) and estimated phytate intake (by tertiles and per each 25 mg/100 kcal).

	**Tertile 1 < 15.0 mg/100 kcal**	**Tertile 2 [15.0 -28.4] mg/100 kcal**	**Tertile 3 > 28.4 mg/100 kcal**	***p*-value for trend**	**Phytate (per 25 mg/100 kcal)**	***p*-value**
**Femoral Neck, n**	185	187	182		554	
T-score ≤ -1, n (%)	96 (51.9)	105 (56.1)	80 (44.0)		281 (50.7)	
Crude Model	1 (ref.)	1.19 (0.79-1.79)	0.73 (0.48-1.10)	0.131	0.77 (0.57-1.03)	0.080
Adjusted Model*	1 (ref.)	1.15 (0.74-1.78)	0.76 (0.48-1.20)	0.237	0.79 (0.57-1.09)	0.144
**Femoral Ward's Triangle, n**	185	187	181		553	
T-score ≤ -1, n (%)	151 (81.6)	146 (78.1)	127 (70.2)		424 (76.7)	
Crude Model	1 (ref.)	0.80 (0.48 - 1.33)	0.53 (0.32-0.86)	0.010	0.62 (0.45-0.87)	0.006
Adjusted Model*	1 (ref.)	0.73 (0.42-1.25)	0.51 (0.30-0.88)	0.014	0.62 (0.43-0.89)	0.010
**Femoral Trochanter, n**	185	187	181		553	
T-score ≤ -1, n (%)	38 (20.5)	41 (21.9)	29 (16.0)		108 (19.5)	
Crude Model	1 (ref.)	1.09 (0.66-1.79)	0.74 (0.43-1.26)	0.279	0.74 (0.50-1.08)	0.121
Adjusted Model*	1 (ref.)	1.03 (0.61-1.74)	0.76 (0.42-1.34)	0.349	0.74 (0.48-1.12)	0.155
**Total Femur, n**	177	184	177		538	
T-score ≤ -1, n (%)	43 (24.3%)	60 (32.6%)	40 (22.6%)		143 (26.6)	
Crude Model	1 (ref.)	1.51 (0.95-2.39)	0.91 (0.56-1.49)	0.718	0.95 (0.68-1.32)	0.741
Adjusted Model*	1 (ref.)	1.42 (0.86-2.34)	0.92 (0.53-1.58)	0.758	0.93 (0.64-1.36)	0.720
**Lumbar Spine L1-L2, n**	148	157	164		469	
T-score ≤ -1, n (%)	91 (61.5)	97 (61.8)	86 (52.4)		274 (58.4)	
Crude Model	1 (ref.)	1.01 (0.64-1.61)	0.69 (0.44-1.08)	0.100	0.74 (0.54-1.01)	0.060
Adjusted Model*	1 (ref.)	0.90 (0.55-1.47)	0.72 (0.44-1.19)	0.190	0.76 (0.54-1.08)	0.127
**Lumbar Spine L1-L3, n**	147	157	164		468	
T-score ≤ -1, n (%)	80 (54.4)	85 (54.1)	68 (41.5)		232 (49.8)	
Crude Model	1 (ref.)	0.99 (0.63-1.55)	0.59 (0.38-0.93)	0.021	0.73 (0.54-1.01)	0.057
Adjusted Model*	1 (ref.)	0.90 (0.55-1.46)	0.61 (0.37-1.00)	0.046	0.76 (0.54-1.07)	0.118
**Lumbar Spine L1-L4, n**	147	156	165		468	
T-score ≤ -1, n (%)	72 (49.0)	78 (50.0)	62 (37.6)		212 (45.3)	
Crude Model	1 (ref.)	1.04 (0.66-1.63)	0.63 (0.40-0.98)	0.039	0.72 (0.53-0.99)	0.045

Adjusted Model*	1 (ref.)	0.95 (0.59-1.55)	0.64 (0.39-1.06)	0.079	0.75 (0.53-1.06)	0.101
**Lumbar Spine L2-L3, n**	167	171	176		514	
T-score ≤ -1, n (%)	90 (53.9)	88 (51.5)	73 (41.5)		251 (48.8)	
Crude Model	1 (ref.)	0.91 (0.59-1.39)	0.61 (0.40-0.93)	0.021	0.71 (0.53-0.97)	0.029
Adjusted Model*	1 (ref.)	0.83 (0.52-1.32)	0.62 (0.39-1.00)	0.048	0.72 (0.52-1.00)	0.052
**Lumbar Spine L2-L4, n**	167	170	177		514	
T-score ≤ -1, n (%)	80 (47.9)	79 (46.5)	66 (37.3)		225 (43.8)	
Crude Model	1 (ref.)	0.94 (0.62-1.45)	0.65 (0.42-0.99)	0.046	0.73 (0.53-0.99)	0.041
Adjusted Model*	1 (ref.)	0.86 (0.54-1.37)	0.64 (0.40-1.03)	0.063	0.7 (20.52-1.01)	0.056
**Lumbar Spine L3-L4, n**	167	170	176		513	
T-score ≤ -1, n (%)	75 (44.9)	71 (41.8)	63 (35.8)		209 (40.7)	
Crude Model	1 (ref.)	0.88 (0.57-1.35)	0.68 (0.44-1.06)	0.086	0.76 (0.56-1.04)	0.087
Adjusted Model*	1 (ref.)	0.81 (0.51-1.28)	0.68 (0.42-1.09)	0.107	0.76 (0.5-1.07)	0.113

Logistic regression models were used to evaluate the association between low BMD and estimated tertiles of phytate intake. Results are expressed as odds ratio (95% CIs). T-score ≤-1 was considered as "low BMD" whereas T-score >-1 was considered "normal BMD.*Models adjusted for age (years), BMI (kg/m2), physical activity (MET•min/week), educational level (higher education/technician or secondary education/primary education or less), smoking status (never/former/current), type 2 diabetes prevalence, osteoporotic fractures prevalence, energy (kcal/day), calcium (mg/day), vitamin D (µg/day), glycemic index, vegetables and fruits (g/day).
