# Supplementary figures and images for: Estimated Phytate Intake Is Associated with Bone Mineral Density in Mediterranean Postmenopausal Women
Source: Nutrients. 2023 Apr 6;15(7):1791. doi: 10.3390/nu15071791 (PMC10097286; doi:10.3390/nu15071791)

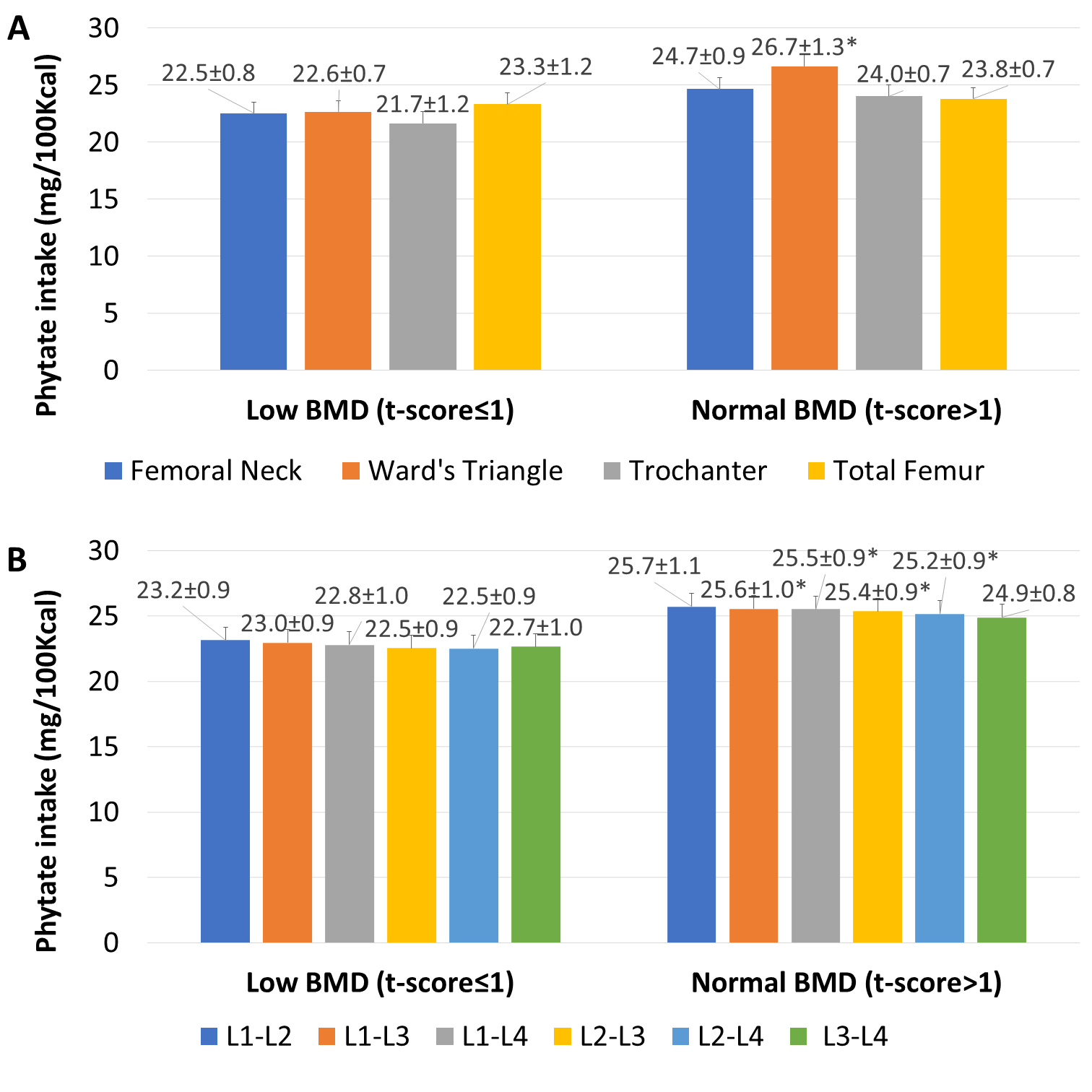

Supplement: Supplementary file 1 [file nutrients-15-01791-s001.zip › Supplementary Figure S1.tif]
